# M6PR- and EphB4-Rich Exosomes Secreted by Serglycin-Overexpressing Esophageal Cancer Cells Promote Cancer Progression

**DOI:** 10.7150/ijbs.79875

**Published:** 2023-01-01

**Authors:** Dongdong Yan, Di Cui, Yun Zhu, Cecilia Ka Wing Chan, Chung Hang Jonathan Choi, Tengfei Liu, Nikki P.Y. Lee, Simon Law, Sai Wah Tsao, Stephanie Ma, Annie Lai Man Cheung

**Affiliations:** 1 School of Biomedical Sciences, Li Ka Shing Faculty of Medicine, University of Hong Kong, Hong Kong SAR, China.; 2 Center for Clinical Big Data and Analytics, the Second Affiliated Hospital, School of Medicine, Zhejiang University, Hangzhou, China.; 3 Department of Biomedical Engineering, The Chinese University of Hong Kong, Hong Kong SAR, China.; 4 Department of Surgery, Li Ka Shing Faculty of Medicine, University of Hong Kong, Hong Kong SAR, China.; 5 The University of Hong Kong - Shenzhen Hospital, Shenzhen, China.

**Keywords:** Exosome, M6PR, EphB4, serglycin, angiogenesis, invasion

## Abstract

Accumulating evidence shows that exosomes participate in cancer progression. However, the functions of cancer cell exosome-transmitted proteins are rarely studied. Previously, we reported that serglycin (*SRGN*) overexpression promotes invasion and metastasis of esophageal squamous cell carcinoma (ESCC) cells. Here, we investigated the paracrine effects of exosomes from *SRGN*-overexpressing ESCC cells (SRGN Exo) on ESCC cell invasion and tumor angiogenesis, and used mass spectrometry to identify exosomal proteins involved. Cation-dependent mannose-6-phosphate receptor (M6PR) and ephrin type-B receptor 4 (EphB4) were pronouncedly upregulated in SRGN Exo. Upregulated exosomal M6PR mediated the pro-angiogenic effects of SRGN Exo both *in vitro* and *in vivo*, while augmented exosomal EphB4 mediated the pro-invasive effect of SRGN Exo on ESCC cells *in vitro.* In addition, *in vitro* studies showed that manipulation of *M6PR* expression affected the viability and migration of ESCC cells. Both M6PR and EphB4 expression levels were positively correlated with that of SRGN in the serum of patients with ESCC. High level of serum M6PR was associated with poor overall survival rates. Taken together, this study presents the first proof that exosomal M6PR and EphB4 play essential roles in tumor angiogenesis and malignancy, and that serum M6PR is a novel prognostic marker for ESCC patients.

## Introduction

Exosomes, a class of nanosized extracellular vesicles, have attracted considerable attention in recent years as novel mediators of a non-classical secretion pathway. The secretion of exosomes is controlled by Rab27a, a member of Rab GTPases 1, and is dependent on calcium 2. In recent years, increasing evidence has validated that exosomes are crucial components of the tumor microenvironment (TME). They work as vehicles to deliver functional molecules, including proteins, messenger RNAs, microRNAs, DNAs and lipids between tumor cells, and between tumor cells and stromal cells, e.g., fibroblasts, endothelial cells and immune cells 3. In terms of proteins borne by exosomes, for example, it was reported that exosomes from colon cancer cells with mutant *KRAS* could promote the invasion of breast cancer cells by transferring amphiregulin (AREG) 4. Hypoxic ovarian cancer cell-derived exosomes can facilitate cancer cell invasion by delivering signal transducer and activator of transcription 3 (STAT3) 5. Exosomes from pancreatic ductal adenocarcinoma deliver macrophage migration inhibitory factor to Kupffer cells which then recruit macrophages to facilitate liver metastasis 6. Exosomal proteins also play an essential role in angiogenesis. E-cadherin on the surface of exosomes isolated from ovarian cancer cells can interact with VE-cadherin on endothelial cells to promote tumor angiogenesis 7. The angiogenesis inhibitor vandetanib was found to increase the secretion of vascular endothelial growth factor-enriched exosomes from endothelial cells, thereby promoting angiogenesis 8. With respect to esophageal cancer, there are currently only two papers on the roles of exosomal proteins in tumor angiogenesis, invasion and metastasis 9, 10, in contrast to the vast amount of literature on the functions of exosomal RNAs 11.

We previously reported that a proteoglycan serglycin (SRGN) is overexpressed in esophageal carcinoma 12 and in highly invasive esophageal squamous cell carcinoma (ESCC) cells lines 13, and that SRGN and its binding partners have autocrine and paracrine tumor-promoting functions in the TME 12, 14. Since exosomes can transfer bioactive molecules between cells, we speculated that they might be involved in mediating the autocrine pro-invasive and paracrine pro-angiogenic function of SRGN. In this study, we investigated the effects of exosomes from *SRGN*-overexpressing ESCC cells on non-transduced ESCC cells and on endothelial cells, and identified differentially expressed exosomal proteins involved in these processes.

## Material and Methods

### Cell lines

Human ESCC cell lines KYSE30, KYSE150, KYSE410 15 from DSMZ (Braunschweig, Germany) and T.Tn 16 were used in this study. KYSE30 was established from a well-differentiated invasive ESCC, while KYSE150 and KYSE410 were derived from poorly-differentiated invasive ESCC. T.Tn was derived from moderately-differentiated ESCC. KYSE150 was the main cell line used because of its short doubling time and high efficiency of lentivirus infection. The other ESCC cell lines served as biological replicates. Luciferase-expressing KYSE150 cells (KYSE150-luc) was used in the lung metastasis experiment as described previously 17. The ESCC cell lines were maintained in RPMI1640 (#R6504, Sigma-Aldrich, St Louis, MO, USA) supplemented with 10% fetal bovine serum (FBS, #10270106, Gibco, Thermo Fisher Scientific, Waltham, MA, USA). All ESCC cell lines were validated by short tandem repeat analysis and tested routinely for mycoplasma contamination. Human umbilical vein endothelial cells (HUVECs, #C-003-5C, Thermo Fisher Scientific) were maintained in Medium 200PRF (Cat. no. M-200PRF-500, Thermo Fisher Scientific) supplemented with 2% of low serum growth supplement (LSGS, #S-003-10, Thermo Fisher Scientific). All cell cultures were kept at 37°C in 5% CO_2_.

### Gene overexpression and silencing

Human ESCC cell lines stably overexpressing *SRGN* and vector control (Con) were established as previously described 12. ESCC cell lines stably overexpressing *M6PR* and vector control (Ctrl) were established by lentiviral infection. The *M6PR* entry clone was synthesized by BGI Tech Solutions (Beijing Liuhe) Co., Limited. (Beijing, China) and the expression clone was constructed using pLenti CMV Puro DEST vector (#17452, Addgene, Watertown, MA, USA) and Gateway™ LR Clonase™ II Enzyme mix (#11791020, Thermo Fisher Scientific). Short hairpin RNAs (shRNAs) against *RAB27A*, *M6PR* and *EPHB4* (**Table S1**) purchased from Sigma-Aldrich, and empty vector pLKO.1 (shCon) were used in stable gene knockdown experiments.

### Collection of conditioned media (CM)

When the cells reached about 50% confluence, the culture medium was replaced by serum-free medium. Forty-eight hours later, the CM was collected, filtered with 0.45 μm filter (#16537-K, Sartorius Stedim Biotech, Goettingen, Germany) to remove the cells, and stored at -80 °C for further use. For Western blot, the CM was concentrated at least 20-fold using Amicon^®^ Ultra - 4 mL Centrifugal Filters Ultracel^®^ - 3K (#UFC800396, Millipore, Billerica, MA, USA).

### Exosome isolation

When cell confluency reached 50%, cells were washed with phosphate-buffered saline (PBS) twice and then cultured for 48 hours in medium supplemented with 10% exosome-depleted FBS (prepared by ultracentrifugation of FBS at 100,000 × g for 18 hours). The CM was then collected for exosome purification. Crude exosomes were isolated by differential centrifugation (DC) as described previously 18. Then the crude exosomes were further purified by OptiPrep^TM^ density gradient ultracentrifugation (DGUC) 19. OptiPrep^TM^ (#D1556, Sigma-Aldrich) was diluted with 0.25 M sucrose in 10 mM Tris (pH 7.5) to generate 5%, 10%, 20% and 40% (w/v) iodixanol solutions. To obtain discontinuous iodixanol gradient, 2.5 mL each of 40, 20 and 10% (w/v) iodixanol solutions and 2 mL of the 5% iodixanol solution were sequentially layered into ultracentrifuge tubes (#332901A, Eppendorf Himac Technologies Co., Ltd., Hitachinaka, Ibaraki, Japan). Crude exosomes in 500 μL PBS was layered on the discontinuous gradient and centrifuged using a P40ST rotor (Eppendorf Himac Technologies Co., Ltd.) for 24 hours at 100,000 × g_avg_ at 4°C. Continuous 10 fractions of 1 mL were taken from the top of the iodixanol gradient. The fractions were then washed in 10 mL PBS or basal culture medium and centrifuged for at 100,000 × g at 4°C. Finally, the pellets were suspended in 200 μL PBS or basal medium without serum for further study. The purified exosomes in fraction 6 were used for Western blot analysis, cell invasion assays and tube formation assays. Nanoparticle tracking analysis (NTA) of purified exosomes was performed using NanoSight NS500 (Malvern Panalytical, Malvern, United Kingdom) and the concentration of exosomes was measured using NS500 or ZetaView^®^ PMX-220 TWIN Laser (Particle Metrix, North Rhine-Westphalia, Germany).

### Imaging of exosomes by transmission electron microscopy (TEM)

Purified exosomes were fixed in 2% paraformaldehyde and then deposited on a formvar-carbon-coated electron microscope grid for 20 min. After washing with PBS, the grid was contrasted in uranyl oxalate (Electron Microscopy Sciences, Hatfield, PA, USA) for 5 min. Air-dried grids were imaged using a Philips CM 100 TEM (Koninklijke Philips N.V., Amsterdam, Netherlands).

### Exosome uptake analysis

Purified exosomes were labeled with PKH26 Red Fluorescent Cell Linker Midi Kit for General Cell Membrane Labeling (#MIDI26, Sigma-Aldrich) according to the manufacturer's manual. The PKH126-labeled exosomes and the control (dyes in PBS) were washed with a large volume of PBS for three times to remove excess dyes. Labeled exosomes and control were then added to HUVECs for 12 hours. After washing twice with PBS, the cells were fixed in 4% paraformaldehyde and stained with 4',6-diamidino-2-phenylindole, dilactate (DAPI, # D-3571, Thermo Fisher Scientific) to visualize the nuclei. The fluorescent signals were examined using a Carl Zeiss LSM 780 confocal microscope system (Zeiss, Jena, Germany).

### Liquid chromatography tandem mass spectrometry (LC-MS/MS)

Exosomes from ESCC cells overexpressing *SRGN* or empty vectors were purified by DGUC. Then exosomes in the 6^th^ fraction (F6) and proteins in the 8^th^ fraction (F8) were lysed in RIPA Lysis and Extraction Buffer (#89900, Thermo Fisher Scientific) supplemented with PhosSTOP™ phosphatase inhibitor cocktail (#04906837001, Roche) and cOmplete™, Mini, ethylenediaminetetraacetic acid-free protease inhibitor cocktail (#04693159001, Roche, Basel, Switzerland). The exosome samples were electrophoresed on polyacrylamide gels and stained with Coomassie brilliant blue (#20278, Thermo Fisher Scientific). The digestion of Coomassie-stained gel bands, LC-MS/MS analysis and data analysis were performed at the Taplin Biological Mass Spectrometry Facility, Department of Cell Biology, Harvard Medical School. A quantitative comparison of the amount of the proteins in each sample was based on the number of the peptides matched to that protein. The differentially expressed proteins (≥ 1.5-fold change) in exosomes isolated from *SRGN*-overexpressing ESCC cells (SRGN Exo) were subjected to Gene Ontology (GO) and pathway enrichment analyses using PANTHER.

### Western blot analysis

Cells or exosomes were lysed in RIPA Lysis and Extraction Buffer supplemented with PhosSTOP™ phosphatase inhibitor cocktail and cOmplete™, Mini, ethylenediaminetetraacetic acid-free protease inhibitor cocktail. The protein concentrations were measured using Pierce™ BCA Protein Assay Kit (#23227, Thermo Fisher Scientific). For Western blot analysis of exosomes, equal number of exosomes from each group was used for comparison. The details of protein extraction and immunoblotting were described previously 20. Information on antibodies used is shown in **Table S2**. Fuji medical x-ray film (#4741023951, Fujifilm, Tokyo, Japan) or Amersham Hyperfilm ECL (#28-9068-39, Chicago, IL, USA), and Clarity Western ECL Substrate (#1705061, Bio-Rad Laboratories) or SuperSignal™ West Femto Maximum Sensitivity Substrate (#34095, Thermo Fisher Scientific) were used for developing. Glyceraldehyde 3-phosphate dehydrogenase (GAPDH) or β-actin was used as loading control for cell lysates.

### Transwell invasion and migration assays

**Figure 1:**
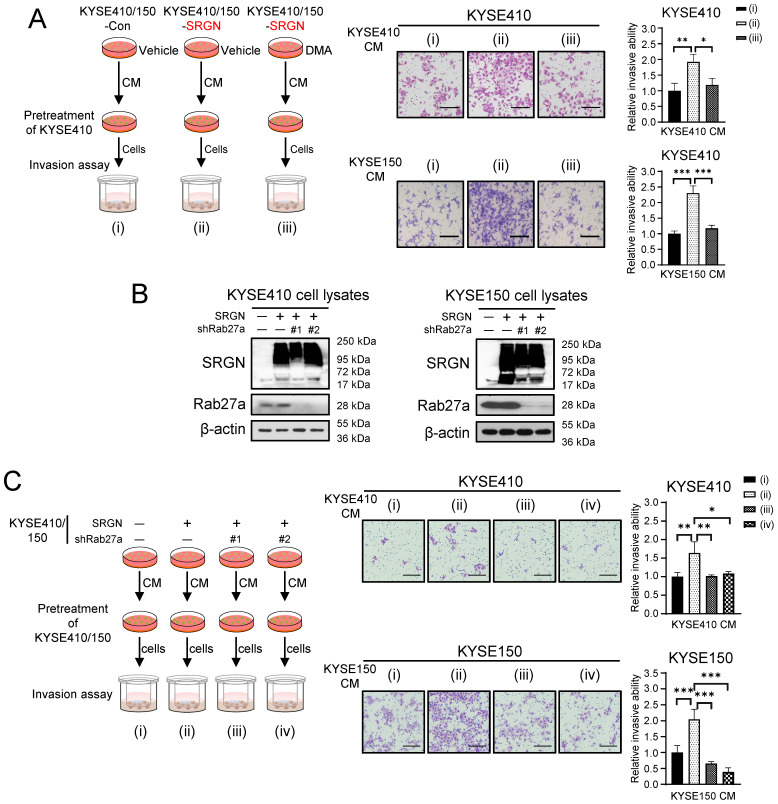
** Inhibition of exosome secretion suppresses pro-invasive effect of SRGN CM on ESCC cells.** (**A**) Effect of DMA treatment on the pro-invasive property of SRGN CM. Left panel, a schematic diagram of the invasion assay; middle panel, representative images of invaded cells; right panel, statistical analysis of the invasion assay. Scale bar, 200 μm. (**B**) Validation of *RAB27A*-knockdown efficiency in ESCC cells with *SRGN* overexpression. Data are presented as mean ± SD. n =3. *, *P* < 0.05; **, *P* < 0.01; ***, *P* < 0.001. (**C**) Effect of *RAB27A-*knockdown on the pro-invasive property of SRGN CM. Left panel, a schematic diagram of the invasion assay; middle panel, representative images of the invasion assay; right panel, statistical analysis of the invasion assay. Scale bar, 200 μm. Data are presented as mean ± SD. n = 4. *, *P* < 0.05; **, *P* < 0.01; ***, *P* < 0.001.

ESCC cells with or without pretreatment with CM or exosomes (1 × 10^9^ exosomes for 1-3 × 10^5^ cancer cells) for 24 hours were suspended in 500 μL serum-free medium and seeded into 8-um pore size inserts (#353097, Corning) coated with 1.2 mg/mL matrigel (#354234, Corning, Corning, NY, USA) for invasion assay. About 700 μL medium containing 10% FBS was placed in the lower chamber as chemoattractant. After 24 hours, the cells on the upper surface of the insert were swept off with cotton swabs and cells that invaded to the lower surface of the insert were stained using 0.1% (w/v) crystal violet (#C0775, Sigma-Aldrich). At least five images were captured for each insert with a 10 × objective lens and the stained cell areas were calculated using ImageJ software. In **Figure 1A**, ESCC cells were treated with 5-(N,N-dimethyl) amiloride hydrochloride (DMA, 100 μM in FBS-free medium, #A4562, Sigma-Aldrich), a known inhibitor of exosome secretion, for 24 hours to inhibit the secretion of exosomes. The medium was then replaced with fresh FBS-free medium which was collected 24 hours later for use in invasion assay. Cell migration assays were conducted as above using inserts without coating.

### Cell viability assay

Resazurin reduction assay was conducted to measure cell viability. In brief, cells were incubated in culture medium containing 0.02% (w/v) resazurin sodium salt (#R7017, Sigma-Aldrich) for 4 hours at 37°C. The fluorescence (570 nm excitation and 600 nm emission) was read on a multilabel plate reader (Varioskan^®^ Flash, #5250040, Thermo Fisher Scientific).

### Endothelial tube formation assay

The endothelial tube formation assay was conducted as described previously 21. Briefly, HUVECs were cultured in Medium 200PRF with 0.4% LSGS for 24 hours and then seeded in a 24-well plate (7 × 10^4^/well) coated with Geltrex™ Reduced Growth Factor Basement Membrane Matrix (#A1413202, Thermo Fisher Scientific). HUVECs were incubated with CM or 2 × 10^8^ exosomes for 6 hours at 37 °C. Six random images were taken using phase contrast microscopy with a 10 × objective lens. The number of nodes, junctions, segments and meshes were quantified using ImageJ software with the Angiogenesis Analyzer plugin 22.

### Quantitative real-time polymerase chain reaction (q-PCR)

Total RNA was extracted using TRIzol (#15596018, Thermo Fisher Scientific) or RNeasy Mini Kit (#74104, Qiagen) following the manufacturers' protocols. Complementary DNA was obtained by using High-Capacity cDNA Reverse Transcription Kit (#4374966, Thermo Fisher Scientific). Q-PCR was conducted by using iTaq universal SYBR green supermix (#1725124, Bio-Rad Laboratories, Hercules, CA, USA). Relative gene expression values were calculated as 2^-ΔΔCt^ which represents the fold change compared with *GAPDH*. The primers used were: 5'-CCACCGGGAAGGTGAATGTC-3' (forward) and 5'-CTGGGCGCACTTTTTGTAGAA-3' (reverse) for human *EPHB4*; 5'-CTGGAGGACTGGACTGCTACT-3' (forward) and 5'-CTCCTACCAAGTCGCAAGTTTT-3' (reverse) for human *M6PR*; 5'-AAGGTCATCCCTGAGCTGAA-3' (forward) and 5'-TGACAAAGTGGTCGTTGAGG-3' (reverse) for human *GAPDH*.

### Tumor xenograft experiment and *in vivo* matrigel plug assay

All animal experiments in this study were approved by the Committee on the Use of Live Animals in Teaching and Research of the University of Hong Kong. About 5 × 10^5^ KYSE150 cells with *SRGN* overexpression or control cells expressing empty vector were suspended in 100 μL PBS-diluted matrigel (final concentration 6 mg/mL) and subcutaneously injected into the right flank of 6-week-old BALB/c female nude mice (n = 6/group). At the endpoint of the experiment, the tumors were dissected for immunohistochemical staining. The *in vivo* matrigel plug assay was performed as previously described 23. Briefly, 300 μL growth factor reduced matrigel (#356231, Corning) containing 6480 U/mL heparin (#H3149, Sigma-Aldrich) and 2 × 10^8^ exosomes or ~ 12 ug/mL human recombinant M6PR (rhM6PR, #TP301277, OriGene, Rockville, MD, USA) was subcutaneously injected into the flank of C57BL/6 mice (n = 4 per group). After 7 days, the matrigel plugs were harvested for hemoglobin quantification using Drabkin's reagent kit (#D5941, Sigma-Aldrich).

### Immunohistochemistry

Immunohistochemistry was conducted on sections of xenograft tumors as previously described 12 using goat anti-CD31 (#sc-1506, Santa Cruz Biotechnology, Dallas, TX, USA) as primary antibody and horseradish peroxidase-linked anti-goat IgG (#MP-7405, Vector Laboratories, Newark, CA, USA) as secondary antibody. The calculation of microvessel density (MVD) was based on CD31 staining as described by Du *et al.* 24. In brief, areas containing the densest CD31-positive microvessels were chosen for counting in sections scanned using a 4 × objective lens. Then MVD was calculated as the average number of microvessels per mm^2^ in the selected area under a 10 × objective lens. A CD31-stained endothelial cell or cluster separate from adjacent vessels was considered as one countable microvessel.

### Experimental metastasis assay

Six-week-old female nude mice (n = 4/group) were primed with 7 × 10^8^ KYSE150-Con Exo or KYSE150-SRGN Exo (suspended in 100 µL PBS) via tail vein injection once a week for 4 weeks. The control group received PBS only. On the 7^th^ day after the last injection, 5 × 10^5^ KYSE150-luc cells were injected via the tail vein. Five weeks later, the mice were intraperitoneally injected with D-luciferin (#LUCK-100, Gold Biotechnology, St Louis, MO, USA) and then subjected to bioluminescence imaging using IVIS Lumina X5 (# CLS148590, Perkin Elmer, Waltham, MA, USA) to assess lung metastasis.

### Enzyme-linked immunosorbent assay (ELISA)

A human M6PR ELISA Kit (#SEH700Hu, Cloud-Clone Corp., Katy, TX, USA) and a human EphB4 IQELISA™ Kit (#IQH-EPHB4-1, RayBiotech, Peachtree Corners, GA, USA) were used to measure the M6PR and EphB4 concentration in the serum samples collected from patients with ESCC at the Queen Mary Hospital (Hong Kong) with approval from the institutional committee for ethical review of research involving human subjects (IRB number: UW19-643). The M6PR and EphB4 levels were correlated with that of SRGN, which was determined in a previous study 12. Kaplan-Meier survival analysis was performed for patients classified into high and low serum M6PR expressions using the median level (20.19 ng/mL) as cut-off value.

### Analysis of gene expression using cancer patient datasets

Gene expressions of *M6PR* in ESCC tumor and the paired tumor-adjacent normal tissues were compared using data from the Gene Expression Omnibus (GEO) database (accession numbers GSE23400 and GSE75241) 25. The *M6PR* expression in several other cancers and normal samples was compared through Gene Expression Profiling Interactive Analysis (GEPIA) using data from The Cancer Genome Atlas (TCGA) and Genotype-Tissue Expression (GTEx) 26.

### Statistical analysis

Statistical analysis was performed using IBM SPSS Statistics 25 (IBM, Chicago, IL, USA). The data collected from *in vitro* experiments were expressed as the mean ± standard deviation of at least three independent experiments. Data from two groups were compared using unpaired or paired Student′s *t*-test, and comparison of data from more than two groups were performed using ordinary one-way analysis of variance (ANOVA). The correlations between SRGN and M6PR, and between SRGN and EphB4 were examined by Pearson's correlation analysis. The overall survival curves were shown as Kaplan-Meier curves and analyzed by Log-rank test. Significant differences were defined when *P* values were less than 0.05 (*, *P* < 0.05; **, *P* < 0.01; ***, *P* < 0.001).

## Results

### Exosomes from *SRGN*-overexpressing ESCC cells promote invasion and metastasis of ESCC cells

To determine if SRGN Exo promote invasion of ESCC cells, two ESCC cell lines with *SRGN*-overexpression (i.e. KYSE410-SRGN and KYSE150-SRGN) and their respective controls expressing empty vectors (i.e. KYSE410-Con and KYSE150-Con) were treated with DMA, an inhibitor of the H^+^/Na^+^ and Na^+^/Ca^2+^ exchangers, to inhibit exosome secretion. The CM were then collected and used to pretreat parental KYSE410 cells in invasion assays (**Figure 1A**). The results showed that the CM of *SRGN*-overexpressing KYSE410 and KYSE150 cells (SRGN CM) had significant pro-invasive effect on parental KYSE410 cells compared with CM of control cells (Con CM), but this effect was abolished by DMA (**Figure 1A**). Since DMA had no adverse effect on the viability of the ESCC cells at the concentration used (**Supplementary Figure S1**), the reduction in pro-invasive effect of CM of DMA-treated ESCC cells (**Figure 1A**) was not due to decreased cell proliferation/viability. Knocking down *RAB27A* using shRNAs (**Figure 1B**) also negated the stimulatory effect of SRGN CM on the invasion of parental ESCC cells (**Figure 1C**).

**Figure 2:**
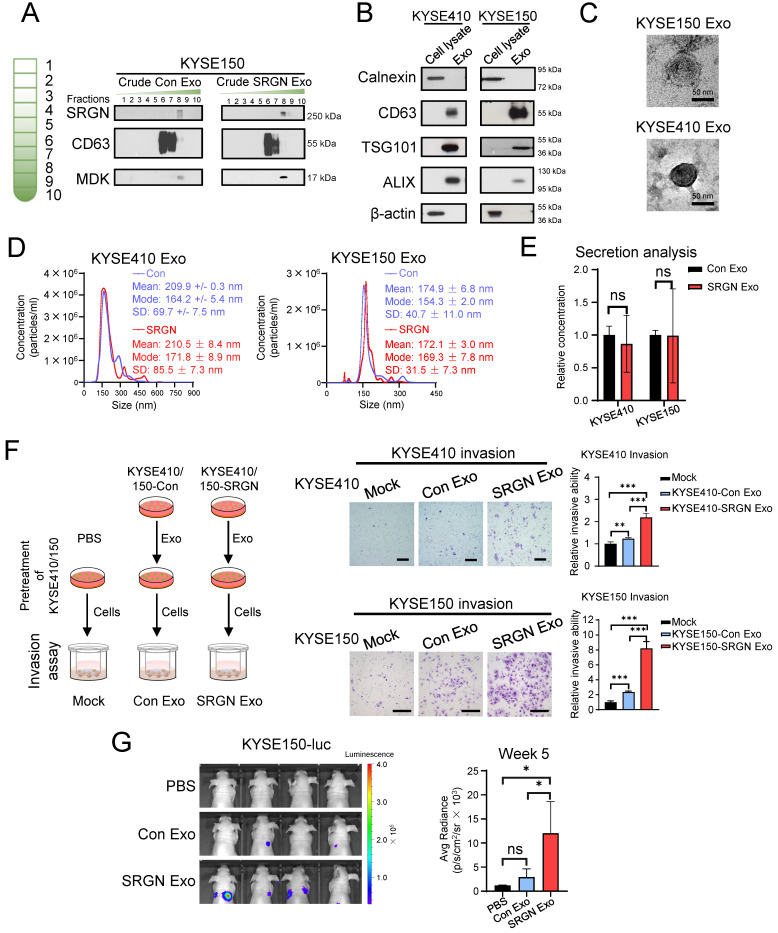
** Exosomes isolated from *SRGN*-overexpressing ESCC cells enhance the invasion and metastasis of parental ESCC cells.** (**A**) Western blot analysis of density gradient fractions of crude exosomes from ESCC cells. Crude exosomes isolated by differential centrifugation were purified by density gradient ultracentrifugation and equal volumes of each fraction were applied for Western blot analysis. (**B-G**) Samples obtained from DUGC F6 were used for further analysis. (**B**) Equal amounts of proteins from cell lysates and exosomes were loaded for comparison by Western blot. (**C**) Representative whole-mount TEM images of exosomes derived from ESCC cells. (**D**) Nanoparticle tracking analysis of exosomes isolated from ESCC cells. (**E**) Quantitative comparison of exosomes secreted by ESCC cells overexpressing *SRGN* and empty vector by ZetaView® PMX-220 TWIN Laser. Data are presented as mean ± SD. n = 3 and 4 for KYSE410 and KYSE150, respectively. ns, not significant. (**F**) Effect of exosomes isolated from Con- and *SRGN*-overexpressing cells on invasion of parental ESCC cells. Left panel, a schematic diagram of the experiment; middle panel, representative images of the invasion assay; right panel, statistical analysis of the invasion assay. Scale bar, 200 μm. Data are presented as mean ± SD. n = 4 and 3 for KYSE410 and KYSE150, respectively. **, *P* < 0.01; ***, *P* < 0.001. (**G**) Effect of SRGN Exo on the colonization of KYSE150-luc cells to lungs of nude mice. Data are presented as mean ± SD. n = 4. ns, not significant; *, *P* < 0.05.

Exosomes were purified from CM of ESCC cells that expressed *SRGN* or empty vector by DC and DGUC. Western blotting showed that exosomes (recognized by anti-CD63) were enriched in DGUC fraction F6 as described in a previous study 19, while SRGN and SRGN-induced midkine (MDK) were mainly found in F8 (**Figure 2A**). The purity of exosomes in DGUC F6 was further assessed by Western blot using cell lysates as control. The exosome markers including CD63, tumor susceptibility gene 101 protein (TSG101) and ALG-2 interacting protein X (ALIX) were detected in the exosomes, whereas calnexin and actin were not (**Figure 2B**). TEM showed that the purified exosomes in DGUC F6 from KYSE410 and KYSE150 cells were round membrane-bound vesicles measuring 57 ± 23 nm and 66 ± 20 nm in diameters, respectively (**Figure 2C**). NTA showed that the size distributions of exosomes from KYSE410 and KYSE150 were similar, and that *SRGN* overexpression did not significantly affect the size of exosomes (**Figure 2D**). These dimensions were consistent with the reported size of desiccated exosomes and hydrated exosomes, respectively, isolated from the serum of a cancer patient 27. NTA also showed that *SRGN* overexpression did not affect the number of exosomes secreted by ESCC cells (**Figure 2E**). Next, parental ESCC cells were incubated with equal number of purified exosomes from corresponding *SRGN*-overexpressing and vector control cells. The results of transwell invasion assay showed that exosomes isolated from control cells (Con Exo) promoted the invasion of parental cells, but SRGN Exo were even more potent (**Figure 2F**). Importantly, *in vivo* experimental metastasis assay showed that pulmonary metastasis of ESCC cells was significantly more pronounced in nude mice primed with circulating SRGN Exo than in the Con Exo group (**Figure 2G**). Taken together, these results showed that SRGN Exo can mediate the transfer of invasive phenotype to other ESCC cells *in vitro* and facilitate metastasis *in vivo*.

### Exosomes from *SRGN*-overexpressing ESCC cells promote angiogenesis *in vitro*

**Figure 3:**
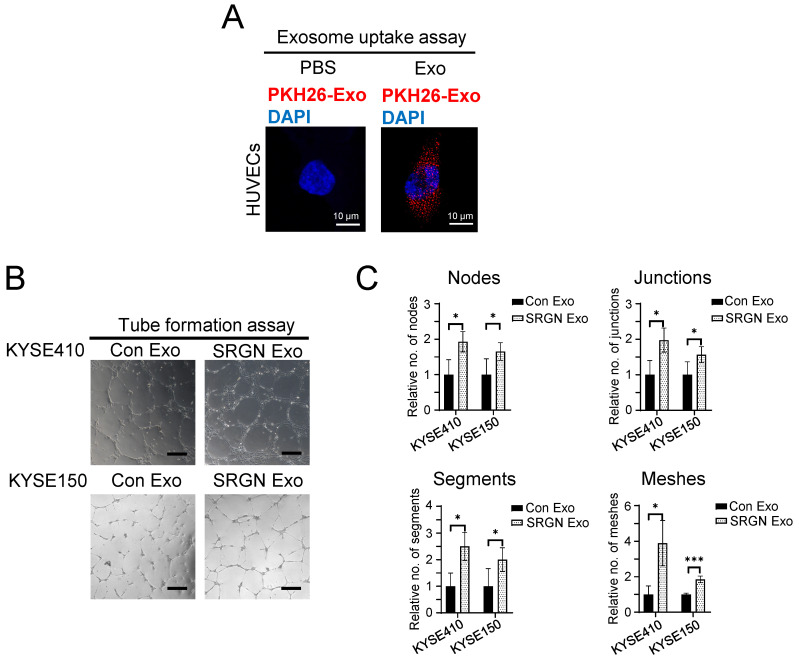
** Exosomes from *SRGN*-overexpressing ESCC cells facilitate angiogenesis *in vitro*.** (**A**) Representative images of uptake of PKH26-labelled exosomes derived from ESCC cells by HUVECs. (**B**) Representative images of HUVECs treated with Con Exo and SRGN Exo from ESCC cells and (**C**) corresponding quantifications of number of nodes, junction, segments and meshes. Scale bar, 200 μm. Data are presented as mean ± SD. n = 3 and 4 for KYSE410 and KYSE150, respectively. *, *P* < 0.05; ***, *P* < 0.001.

Angiogenesis is required to support tumor growth. We found that tumor xenografts of *SRGN*-overexpressing ESCC cells showed increased CD31-positive MVD (**supplementary Figure S2**). To determine if SRGN Exo had pro-angiogenic effects, we first ascertained that purified exosomes from ESCC cells could be taken up by endothelial cells by incubating PKH26-labeled exosomes with HUVECs (**Figure 3A**). *In vitro* angiogenesis assay was then performed, and the results showed that SRGN Exo enhanced the tube forming ability of HUVECs, which was indicated by the increased numbers of nodes, junctions, segments and meshes (**Figure 3B, C**).

### Cation-dependent mannose-6-phosphate receptor (M6PR) and ephrin type-B receptor 4 (EphB4) are enriched in exosomes purified from *SRGN*-overexpressing ESCC cells

**Figure 4:**
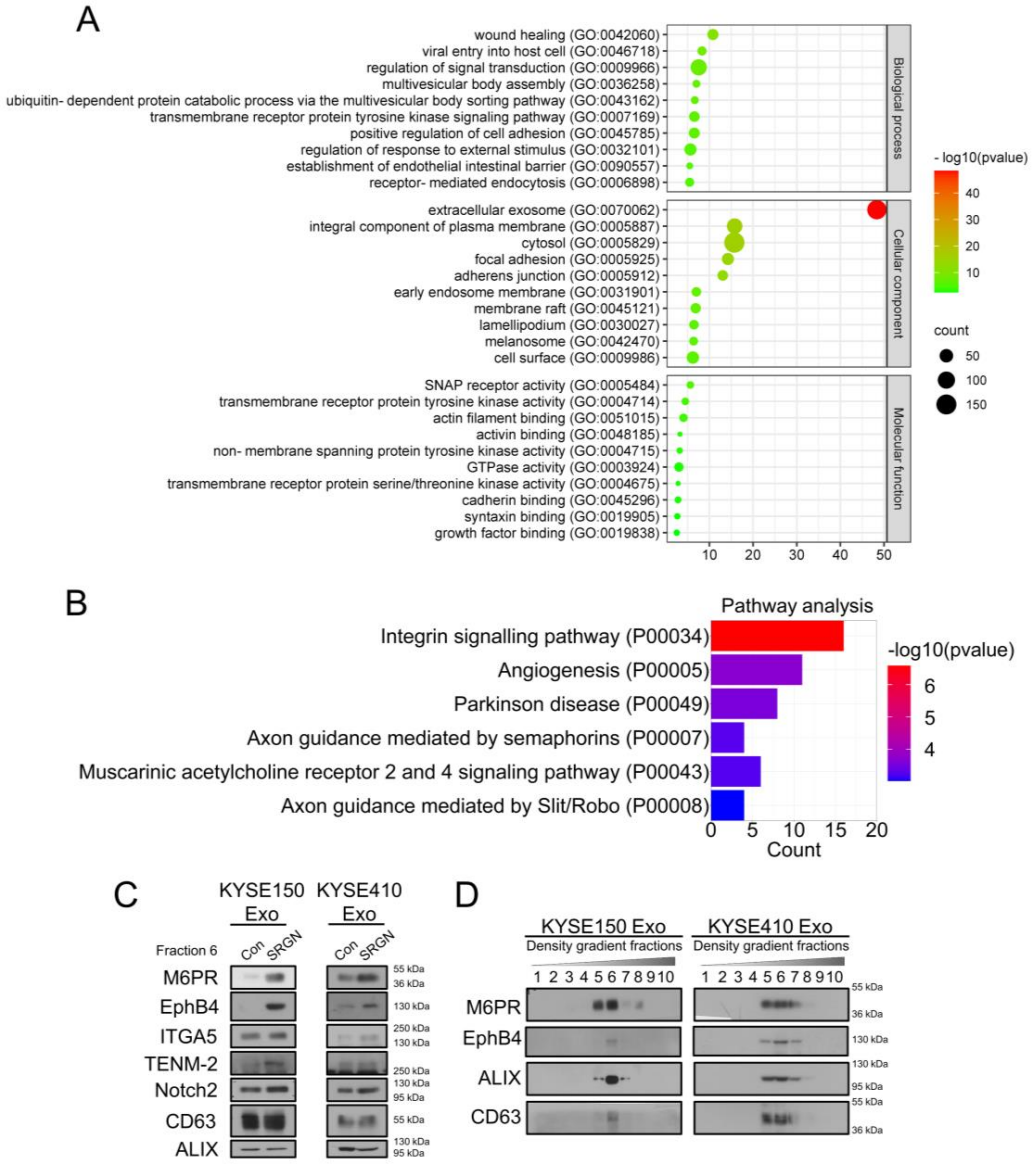
** M6PR and EphB4 are enriched in exosomes from *SRGN*-overexpressing ESCC cells.** (**A**) GO analysis of differentially expressed proteins in exosomes from KYSE150-SRGN. (**B**) PANTHER™ Pathway enrichment analysis for differentially expressed proteins in exosomes from KYSE150-SRGN. All enriched pathways were shown. (**C**) Western blot validation of the upregulated proteins identified by LC-MS/MS in SRGN Exo compared with Con Exo. The concentration of exosomes was measured by Nanosight NS500 and equal numbers of exosomes were used for Western blot analysis. (**D**) Western blot analysis of M6PR, EphB4, ALIX and CD63 expressions in density gradient fractions of exosomes from ESCC cells. After flotation of crude exosomes in iodixanol gradients, equal volumes of each fraction were used for Western blot analysis.

Our previous study showed that SRGN-induced MDK, which is a known angiogenic factor, mediates the pro-invasive effect of SRGN 12. Since fractions near DGUC F8 were reported to contain soluble proteins 28, and **Figure 2A** showed that SRGN and SRGN-induced MDK were detected in DGUC F8 rather than F6, SRGN and MDK were unlikely to be carried by exosomes. To identify proteins that mediate the pro-invasive effect of SRGN Exo, proteins in the DGUC F6 fractions of KYSE150-SRGN Exo and KYSE150-Con Exo were analyzed using LC-MS/MS (**Table S3**). To ensure that the data were not confounded by non-exosomal proteins, the SRGN-induced proteins which were ≥1.5 more abundant in DGUC F8 than in F6 were excluded from that of DGUC F6 for subsequent analyses. GO analysis of differentially expressed proteins (≥ 1.5-fold change) in SRGN Exo showed that these proteins were associated with regulation of signal transduction, multivesicular body assembly and establishment of endothelial intestinal barrier in the biological process category, extracellular exosomes in the cellular component category, and soluble N-ethylmaleimide-sensitive fusion protein attachment protein (SNAP) receptor activity and GTPase activity in the molecular function category (**Figure 4A**). PANTHER™ Pathway enrichment analysis showed that the differentially expressed proteins in SRGN Exo were associated with integrin signaling pathway and angiogenesis (**Figure 4B**). Proteins with ≥1.5 fold upregulation and ≥6 peptides in SRGN Exo F6 were listed in **Table 1**. The top upregulated proteins including teneurin-2 (TENM2), growth factor receptor bound protein 2 (GRB2), lectin mannose binding 1 (LMAN1), stromal cell derived factor 4 (SDF4), Golgi membrane protein 1 (GOLM1), M6PR and integrin α-5 (ITGA5), as well as two other proteins including EphB4 and neurogenic locus notch homolog protein 2 (Notch2) which were reported to be associated with cancer invasion 29, 30 were subjected to validation by Western blot. The results confirmed elevation of M6PR, EphB4, ITGA5, TENM-2 and Notch2 in SRGN Exo, but only M6PR and EphB4 were consistently and markedly upregulated in multiple ESCC cell lines (**Figure 4C**). The expression levels of GRB2, LMAN1, SDF4 and GOLM1 in SRGN Exo were, however, either lower or similar to that of Con Exo (**Supplementary Figure S3**). To further confirm that M6PR and EphB4 were expressed in exosomes, crude exosomes isolated from two ESCC cell lines by DC were separated into different fractions by DGUC for Western blot analysis. The results confirmed that M6PR and EphB4 were indeed enriched in the same fractions as the exosome markers ALIX and CD63 (**Figure 4D**), and therefore carried by exosomes.

### M6PR is upregulated in ESCC and has prognostic significance

**Figure 5:**
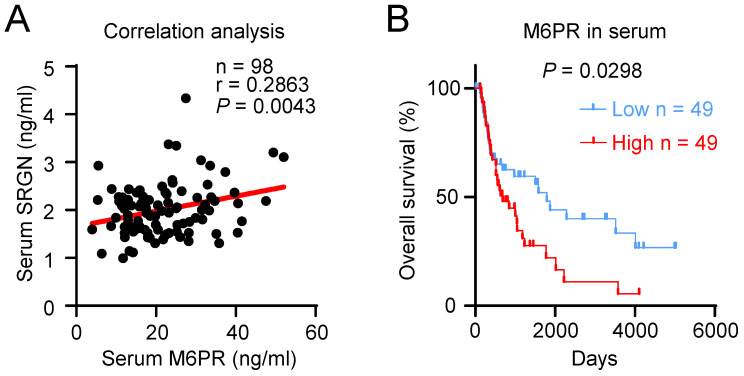
** M6PR has prognostic significance in ESCC patients.** (**A**) Correlation analysis between serum SRGN and M6PR in 98 patients with ESCC. (**B**) Kaplan-Meier curves comparing the survival outcome of ESCC patients with high versus low serum M6PR expression.

Data from TCGA and GTEx showed that a variety of human cancers expressed a higher level of *M6PR* mRNA (**Supplementary Figure S4A**). Analysis of data from GEO datasets (GSE23400 and GSE75241) showed that the *M6PR* expression level was higher in ESCC compared with the adjacent normal tissue (**Supplementary Figure S4B**). However, q-PCR analysis showed that *SRGN* overexpression did not significantly increase *M6PR* mRNA expression in ESCC cells (**Supplementary Figure S4C**), which suggests that SRGN regulates the secretion of M6PR. ELISA of serum samples of patients with ESCC showed that M6PR and SRGN expression levels were positively correlated (**Figure 5A**). Notably, Kaplan-Meier survival analysis indicated that high serum M6PR was significantly associated with poor overall survival rates (**Figure 5B**).

### Exosomal M6PR mediates the pro-angiogenic function of SRGN Exo

**Figure 6:**
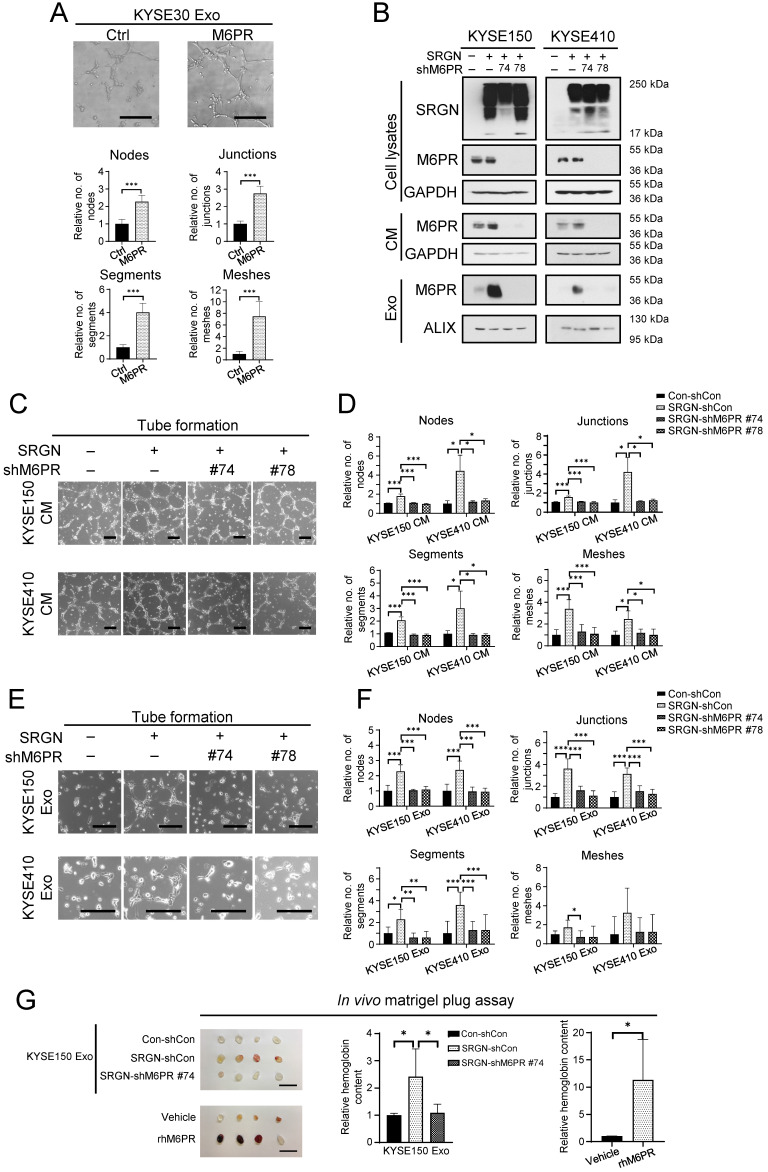
** Exosomal M6PR mediates the effect of SRGN on angiogenesis *in vitro* and *in vivo*.** (**A**) Effect of M6PR Exo on tube formation ability of HUVECs. Scale bar, 200 μm. Data are presented as mean ± SD. n = 7. ***, *P* < 0.001. (**B**) Western blot analysis of M6PR in cell lysates, CM and exosomes of ESCC cells with manipulated *SRGN* and *M6PR* expression. (**C**) Representative images of HUVECs treated with indicated CMs from ESCC cells. Scale bar, 200 μm. (**D**) Quantification of the numbers of nodes, junctions, segments and meshes in (C). Data are presented as mean ± SD. n = 8 and 3 for KYSE150 and KYSE410, respectively. *, *P* < 0.05; ***, *P* < 0.001. (**E**) Representative images of HUVECs treated with indicated Exo from ESCC cells. Scale bar, 200 μm. (**F**) Quantification of the numbers of nodes, junctions, segments and meshes in (E). Data are presented as mean ± SD. n = 6. *, *P* < 0.05; **, *P* < 0.01; ***, *P* < 0.001. (**G**) Effects of exosomes from ESCC cells with *SRGN* overexpression and *M6PR*-knockdown and rhM6PR on *in vivo* angiogenesis. Data are presented as mean ± SD. n = 4. *, *P* < 0.05.

Overexpression and knockdown experiments showed that although M6PR regulated ESCC cell viability and cell migration (**Supplementary Figure S5A-E**), neither *M6PR* expression *per se* nor M6PR-enriched exosomes isolated from *M6PR*-expressing ESCC cells had pro-invasive effects on ESCC cells (**Supplementary Figure S5F, G**). However, results from *in vitro* angiogenesis assay showed that M6PR-rich exosomes promoted tube formation of HUVECs (**Figure 6A**). Furthermore, knocking down *M6PR* in *SRGN*-overexpressing ESCC cells, which resulted in a marked decrease in M6PR in cell lysate, CM and exosomes (**Figure 6B**), abolished the stimulatory effect of both SRGN CM and SRGN Exo on tube formation of HUVECs (**Figure 6C-F**). Matrigel plug assay was conducted to evaluate the function of SRGN Exo and rhM6PR in angiogenesis *in vivo*. The results showed that they both facilitated neovascularization in cell-free matrigel plugs, as indicated by the significantly higher hemoglobin content (**Figure 6G**). Importantly, SRGN Exo lost their pro-angiogenic property after *M6PR*-knockdown (**Figure 6G**). These data suggest that cancer cell-secreted M6PR-rich exosomes are important angiogenic mediators in *SRGN*-overexpressing ESCC.

### Exosomal EphB4 partially mediates the pro-invasive effect of SRGN Exo on non-transduced ESCC cells

**Figure 7:**
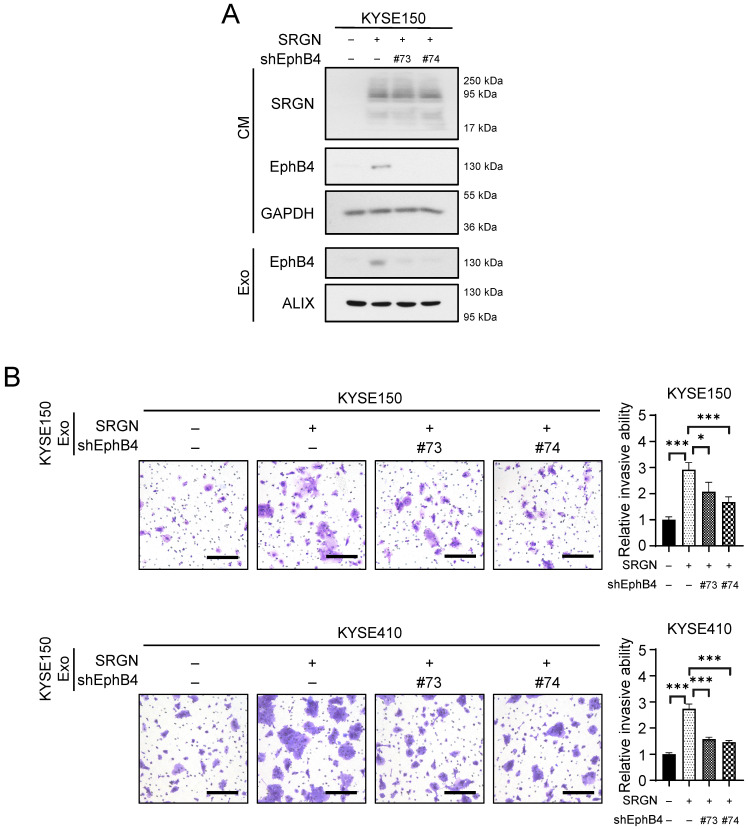
** Exosomal EphB4 partially mediates the effect of SRGN on invasion of ESCC cells.** (**A**) Validation of *EPHB4*-knockdown efficiency in ESCC cells with SRGN overexpression. (**B**) Effect of *EPHB4*-knockdown on pro-invasive ability of exosomes derived from SRGN-overexpressing ESCC cells. Scale bar, 200 μm. Data are presented as mean ± SD. n = 4. *, *P* < 0.05; ***, *P* < 0.001.

Since M6PR-rich Exo did not facilitate the invasion of ESCC cells (**Supplementary Figure S5G**), we examined whether the other obviously upregulated protein in SRGN Exo, i.e. EphB4, which was positively correlated with SRGN in the serum of patients with ESCC (**Supplementary Figure S6**), mediated the pro-invasive effect of SRGN. Knockdown of *EPHB4* in *SRGN*-overexpressing ESCC cells by shRNAs markedly reduced EphB4 expression in the CM and exosomes purified by DC and DGUC (**Figure 7A**). The results of invasion assays showed that *EPHB4*-knockdown attenuated the pro-invasive effect of SRGN Exo on ESCC cells (**Figure 7B**).

## Discussion

SRGN was reported to promote cancer progression by facilitating the invasion 12, vascularization 31, metastasis 12, 32 and chemoresistance 33 of cancer cells. In this study, the results suggest that SRGN Exo could mediate the influence of more invasive ESCC cells on less invasive ESCC cells. Surprisingly, the pro-invasive effect of SRGN CM was almost completely abolished by DMA or *RAB27A*-knockdown (**Figures 1A** and** 1C**). A possible explanation is that, in addition to exosome inhibition, DMA treatment and *RAB27A*-silencing might have other effects that further reduced the pro-invasive property of SRGN CM. For instance, DMA inhibits the secretion of exosomes by decreasing the intracellular Na^+^ and Ca^2+^ 2, 34, but changes in intracellular Ca^2+^ also affects protein secretion 35 and the expression of matrix metalloproteinase 2 (MMP2) 36 and MMP9 37. It was also reported that *RAB27A*-knockdown decreased the secretion of MMP9 from breast cancer cells 38, 39. Data from our previous study showed that MMP2 and MMP9 were increased in the SRGN CM 12. Since the mass spectrometry results showed that MMP2 and MMP9 were absent in SRGN Exo and Con Exo (**Table S3**), it is possible that decreased secretion of non-exosome-associated matrix-degrading MMPs from *SRGN*-overexpressing ESCC cells had contributed to the strong suppressive effects of DMA and *RAB27A*-knockdown on ESCC cell invasion observed in **Figure 1**.

Although several mass spectrometry studies showed the presence of SRGN in exosomes 40-42, functional validation of exosomal SRGN was performed in only one study in which it was found to play a key role in regulating the protein cargo of exosomes in human myeloma cells 43. In the present study, overexpression of *SRGN* in ESCC cells altered the protein profile of exosomes, and endowed the SRGN Exo with the capability to promote cancer progression through autocrine and paracrine mechanisms. The results of GO analysis showed that differentially expressed proteins in SRGN Exo were associated with GO terms such as multivesicular body assembly and SNAP receptor activity, which are relevant to exosome biogenesis 3, 44, 45. In terms of cellular components, the most significant GO term was extracellular exosome, which attested to both the purity of exosomes obtained by DGUC and the effect of SRGN on the formation of exosomes. However, data from DGUC and Western blotting showed that SRGN expression was negligible in the exosomes of ESCC cells (**Figure 2A**), which suggested that the pro-invasive and pro-angiogenic functions of SRGN Exo were likely to be mediated by exosomal molecules other than SRGN itself. Interestingly, not all SRGN-induced secreted proteins were enriched in the exosomes. Besides MMP2 and MMP9 discussed above, MDK 12 and interleukin-1β (IL-1β) 14 were also not detected in SRGN Exo of ESCC cells (**Table S3**) even though MDK was found in exosomes derived from melanoma cells 46 and neuroblastoma cells 47. Taken together, our findings suggest that the exosomes may be responsible for transporting a distinct subset of functional proteins to target cells.

M6PR was found to be the most abundant protein (in terms of the number of total peptides) among the top 10 most upregulated proteins in SRGN Exo. The function of M6PR in ESCC had not been explored previously. Here, novel data are presented to show that M6PR facilitates viability and migration of ESCC cells. Furthermore, although previous mass spectrometry studies indicated that M6PR was present in exosomes from endothelial cells 48 and various types of cancer cells, such as chronic B cell leukemia cells 42, T cell lymphoma cells 49 and ovarian cancer cells 50, further validation and functional study of exosomal M6PR were not performed. The present study provides the first evidence of the presence of M6PR in exosomes from ESCC cells. This finding is relevant to previous studies showing that M6PR was present in endosomes 51 from which exosomes are formed. The mechanism of M6PR secretion has never been studied. M6PR carries newly synthesized acid hydrolases from *trans*-Golgi network to endosomes, and is recycled back to the *trans*-Golgi network 52. If the return of M6PR to the *trans*-Golgi network is attenuated, it may become concentrated in the endosomes, and subsequently accumulate in endosome-derived exosomes in the extracellular space. It is worth further study to uncover whether SRGN affects the return of M6PR into the Golgi. It was reported that M6PR may reach the plasma membrane as a result of mis-sorting 52. It is possible that SRGN can enhance this mis-sorting, and therefore increase the secretion of M6PR carried by microvesicles which are formed through budding of the plasma membrane.

Even though exosomes isolated from *M6PR*-overexpressing ESCC cells did not facilitate invasion of ESCC cells, they were highly potent in stimulating tube formation of endothelial cells. The data in **Figure 6** showed that SRGN facilitates angiogenesis by upregulating exosome-delivered M6PR. Tumor angiogenesis involves the following steps: (1) endothelial cell activation and extracellular matrix (ECM) degradation upon induction by angiogenic stimulus; (2) endothelial cell invasion, sprouting and proliferation in ECM; (3) sprout fusion, vessel lumen and network formation; (4) vessel maturation and stabilization by new ECM synthesis and pericyte recruitment 53-55. The increase in the numbers of nodes, junctions, segments and meshes in the *in vitro* tube formation assay suggested that exosomal M6PR may be associated with the processes of endothelial cell sprouting and network formation.

The mechanism by which exosomal M6PR facilitates angiogenesis is still not clear. It is well known that M6PR can deliver acid hydrolases to endosomes and then to lysosomes, which is essential for the function of lysosomes 56. Exosomes and lysosomes are both derived from multivesicular bodies which is a subset of endosomes 57, 58. Multivesicular bodies can fuse with plasma membrane to release exosomes and fuse with lysosomes to provide newly synthesized lysosomal proteins. It was reported that changes of lysosome function affect the secretion of exosomes 59. It is also possible that treatment with exosomes may affect the function of lysosomes in a M6PR-dependent manner. After M6PR-rich exosomes are taken up by endothelial cells, more M6PR may be incorporated into endosomes which belong to the delivering system of lysosomal proteins and finally enhance the function of lysosomes. Furthermore, it was reported that lysosomes promote angiogenesis by releasing cathepsins to facilitate degradation of vascular basement membrane and ECM 60, 61, regulate endothelial cell migration 62, and produce cholesterol to enhance angiogenic signaling 63. Taken together, exosomal M6PR may enhance the function of lysosomes in endothelial cells to facilitate angiogenesis.

Eph receptors and their ligands, ephrins, are both expressed on the cell surface and play an essential role in intercellular communication during cancer progression 64. The binding of Eph receptors with ephrins can trigger bidirectional signaling pathways: forward signaling pathways that are dependent on Eph kinase activity and spread in the receptor-expressing cells, and reverse signaling pathways that are dependent on Src family kinases and spread in the ephrin-expressing cells. Both EphB4 and its preferred ligand, ephrin-B2, are overexpressed in ESCC 65, 66. Moreover, it was reported that ephrin type-B receptor 2 (EphB2) carried by small extracellular vesicles stimulated ephrin-B reverse signaling in endothelial cells 67. Therefore, it is possible that exosomal EphB4 can promote the invasion of ESCC cells by binding with ephrin-B2 and activating reverse signaling pathway. Since exosomes are formed by double invagination of the plasma membrane 3, it is possible that EphB4 on plasma membrane is loaded into exosomes via endocytosis. As *SRGN* overexpression did not obviously upregulate *EPHB4* mRNA in ESCC cells (**Supplementary Figure S7**), the pronounced increase of extracellular EphB4 (**Figure 4**) might be due to enhanced secretion via exosomes. How SRGN facilitates EphB4 loading into exosomes needs further exploration. Because SRGN can enhance the secretion of many growth factors and cytokines 68, and the binding of ligands to receptors can initiate endocytosis 69, we speculate that certain ligands induced by SRGN may increase endocytosis and EphB4 loading into exosomes.

In conclusion, the findings in this study demonstrated that exosomes from *SRGN*-overexpressing ESCC cells play important roles in cancer progression, with SRGN- induced exosomal M6PR and EphB4 having pro-angiogenic and pro-invasive functions, respectively. The importance of M6PR in ESCC was further evidenced by gene expression analysis of GEO datasets which showed that it is overexpressed in ESCC, functional assays showing its regulatory function on migration and viability of ESCC cells, as well as its potential as a serum prognostic marker.

## Figures and Tables

**Table 1:** Top upregulated proteins in SRGN Exo (identified by LC-MS/MS) and their peptide numbers compared with that of Con Exo

	Number of total peptides		Fold Change
	Con Exo	SRGN Exo	
TENM2	0.01	9	900
GRB2	0.01	9	900
LMAN1	0.01	8	800
SDF4	0.01	6	600
GOLM1	1	7	7.0
M6PR	6	19	3.2
ITGA5	3	8	2.7
COL1A1	4	9	2.3
VPS25	4	8	2.0
SERINC5	4	8	2.0
RTN4	3	6	2.0
GNG12	5	9	1.8
GSTP1	6	10	1.7
IMPAD1	5	8	1.6
EPHB4	5	8	1.6
TOM1L1	14	21	1.5
STX4	10	15	1.5
LMAN2	6	9	1.5
YKT6	6	9	1.5
PFN1	6	9	1.5
SDC1	4	6	1.5
NOTCH2	4	6	1.5
SCARB1	4	6	1.5
EFNB2	4	6	1.5

## Abbreviations

ALIX: ALG-2 interacting protein X
ANOVA: analysis of variance
AREG: amphiregulin
CM: conditioned medium
Con/Ctrl: vector control
Con CM: CM of cells overexpressing empty vectors
Con Exo: exosomes isolated from cells overexpressing empty vectors
DAPI: 4',6-diamidino-2-phenylindole, dilactate
DC: differential centrifugation
DGUC: density gradient ultracentrifugation
DMA: 5-(N,N-dimethyl) amiloride hydrochloride
ECM: extracellular matrix
ELISA: enzyme-linked immunosorbent assay
EphB2: ephrin type-B receptor 2
EphB4: ephrin type-B receptor 4
ESCC: esophageal squamous cell carcinoma
FBS: fetal bovine serum
GAPDH: glyceraldehyde 3-phosphate dehydrogenase
GEO: Gene Expression Omnibus
GEPIA: Gene Expression Profiling Interactive Analysis
GO: Gene Ontology
GOLM1: Golgi membrane protein 1
GRB2: growth factor receptor bound protein 2
GTEx: Genotype-Tissue Expression
HUVECs: human umbilical vein endothelial cells
IL-1β: interleukin-1β
ITGA5: integrin α-5
LC-MS/MS: liquid chromatography tandem mass spectrometry
LMAN1: lectin mannose binding 1
LSGS: low serum growth supplement
M6PR: mannose-6-phosphate receptor
MDK: midkine
MMP: matrix metalloproteinase
MVD: microvessel density
Notch2: neurogenic locus notch homolog protein 2
NTA: nanoparticle tracking analysis
PBS: phosphate-buffered saline
q-PCR: quantitative real-time polymerase chain reaction
SDF4: stromal cell derived factor 4
shRNA: short hairpin RNA
SRGN: serglycin
SRGN CM: CM of SRGN-overexpressing ESCC cells
SRGN Exo: exosomes isolated from *SRGN*-overexpressing ESCC cells
STAT3: signal transducer and activator of transcription 3
TENM2: teneurin-2
TEM: transmission electron microscopy
TME: tumor microenvironment
TSG101: tumor susceptibility gene 101 protein
