# Supplementary material for: M6PR- and EphB4-Rich Exosomes Secreted by Serglycin-Overexpressing Esophageal Cancer Cells Promote Cancer Progression
Source: Int J Biol Sci. 2023 Jan 1;19(2):625–40. doi: 10.7150/ijbs.79875 (PMC9830512; doi:10.7150/ijbs.79875)
Supplement: Supplementary file 1 — Supplementary figures and tables 1-2. [file ijbsv19p0625s1.pdf]

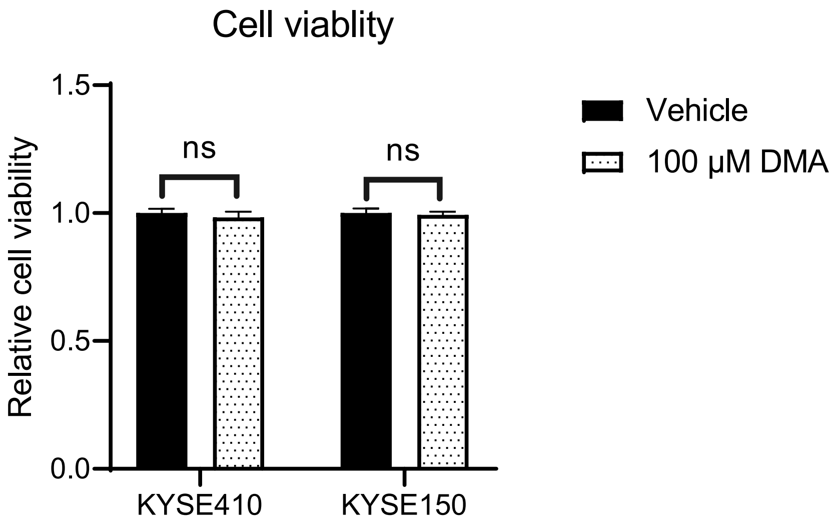

Figure S1. Effect of DMA treatment on cell viability of ESCC cells. The cell viability of ESCC cells was examined 24 hours after treatment with DMA (100 μM in FBS-free medium). Data are presented as mean ± SD. n = 3. ns, not significant.

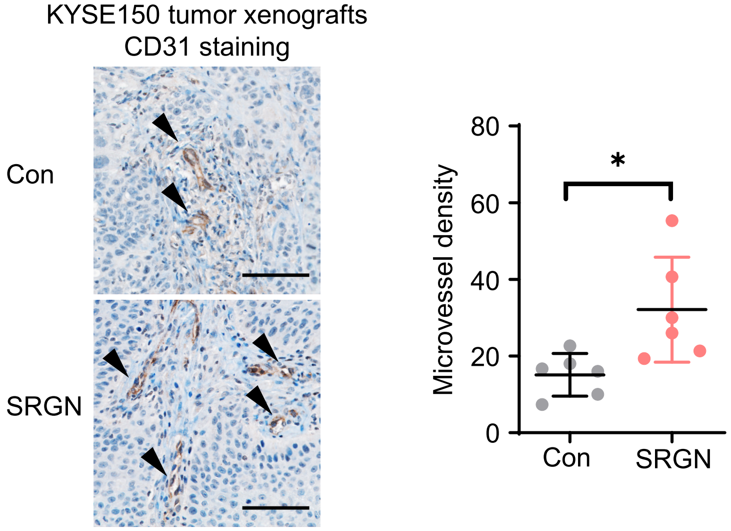

Figure S2. Effect of *SRGN* overexpression in ESCC cells on tumor angiogenesis. Left panel, representative images of CD31-immunostained sections of tumor xenografts; scale bar, 100 μm. Right panel, the corresponding quantifications of microvessel density. Data are presented as mean ± SD. n = 6. *, $P < 0.05$.

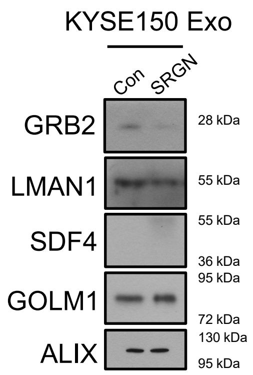

Figure S3. Analysis of GRB2, LMAN1, SDF4 and GOLM1 expressions in SRGN Exo and Con Exo. The concentration of exosomes was measured by Nanosight NS500 and equal numbers of exosomes were used.

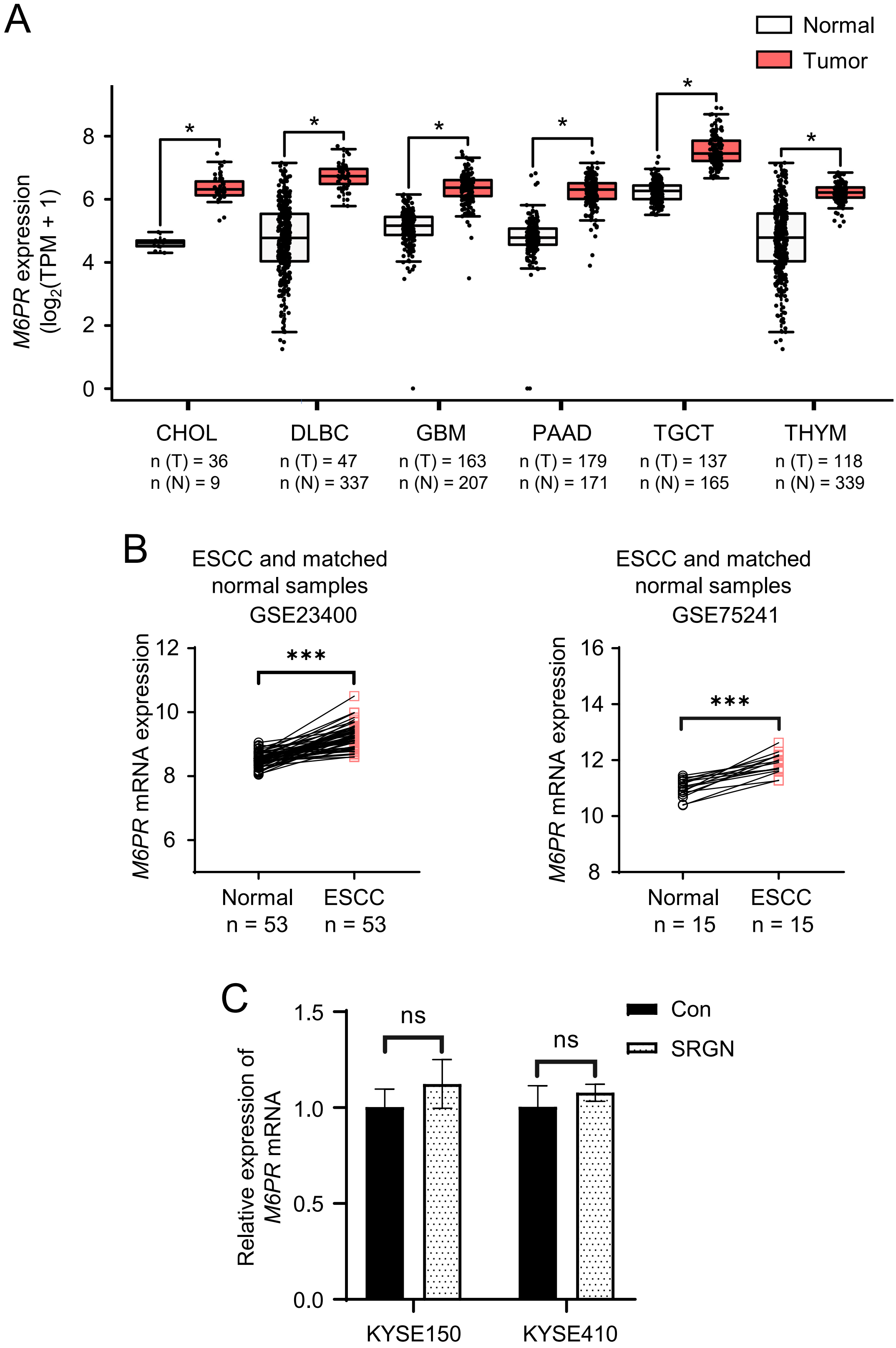

Figure S4. ***M6PR*** **expression in human tumors and effect of** ***SRGN*** **overexpression on** ***M6PR*** **mRNA expression in ESCC cells.** (A) Comparison of *M6PR* expression in human tumors and normal samples using data from TCGA through GEPIA. CHOL, cholangio carcinoma; DLBC, lymphoid neoplasm diffuse large B-cell lymphoma; GBM, glioblastoma multiforme; PAAD, pancreatic adenocarcinoma; TGCT, testicular germ cell tumors; THYM, thymoma. TPM, transcripts per million. *, $P < 0.05$. (B) Comparison of *M6PR* mRNA expression level in human ESCC tissue samples and matched normal tissue samples using data from The Gene Expression Omnibus database (GSE23400 and GSE75241). ***, $P < 0.001$. Paired Student's *t*-test. (C) Q-PCR analysis of the effect of *SRGN* overexpression on *M6PR* mRNA expression in KYSE150 and KYSE410 cells. Data are presented as mean ± SD. n = 3. ns, not significant.

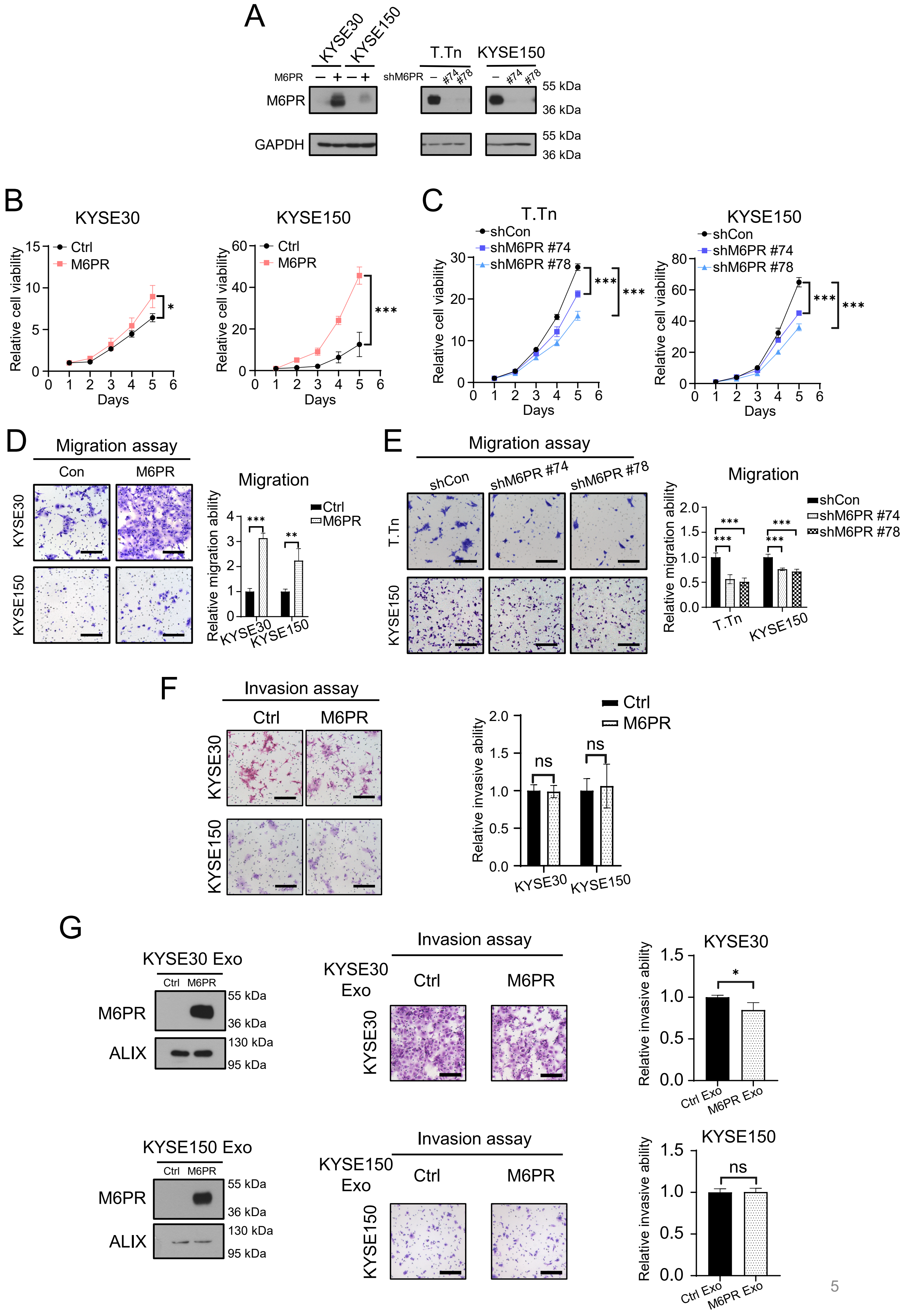
A
KYSE30
KYSE150
M6PR
M6PR
GAPDH
T.Tn
KYSE150
shM6PR
#74
#78
55 kDa
36 kDa
B
KYSE30
KYSE150
Ctrl
M6PR
Relative cell viability
Days
C
T.Tn
KYSE150
shCon
shM6PR #74
shM6PR #78
D
Migration assay
Con
M6PR
Migration
Relative migration ability
E
Migration assay
shCon
shM6PR #74
shM6PR #78
F
Invasion assay
Ctrl
M6PR
Relative invasive ability
ns
G
KYSE30 Exo
KYSE150 Exo
ALIX
130 kDa
95 kDa
Invasion assay
Ctrl Exo
M6PR Exo

Figure S5. *In vitro* functional effects of M6PR on ESCC cells. (A) Western blot validation of *M6PR* overexpression and knockdown using ESCC cell lysates. (B) The effect of *M6PR* overexpression on viability of ESCC cells. Data are presented as mean ± SD. n = 3. *, $P < 0.05$; ***, $P < 0.001$. (C) The effect of *M6PR* knockdown on viability of ESCC cells. Data are presented as mean ± SD. n = 3. ***, $P < 0.001$. (D-E) Transwell migration assay of ESCC cells with *M6PR* overexpression (D) or *M6PR* knockdown (E). Scale bar, 200 μm. Data are presented as mean ± SD. n = 4 in (D) and n = 4 and 5 for T.Tn and KYSE150 in (E). **, $P < 0.01$; ***, $P < 0.001$. (F) Transwell invasion assay of ESCC cells with *M6PR* overexpression. Scale bar, 200 μm. Data are presented as mean ± SD. n = 3. ns, not significant. (G) Effect of exosomal M6PR on invasion of ESCC cells. Scale bar, 200 μm. Data are presented as mean ± SD. n = 4. ns, not significant; *, $P < 0.05$.

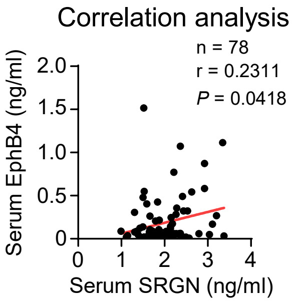

Figure S6. Correlation analysis between serum SRGN and EphB4 in 78 patients with ESCC.

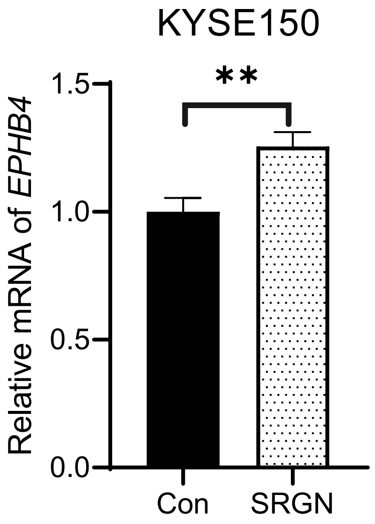

Figure S7. Q-PCR analysis of the effect of SRGN overexpression on EPHB4 mRNA expression in KYSE150. Data are presented as mean ± SD. n = 3. **, $P < 0.01$.

Supplementary tables

Table S1. Oligonucleotide sequences of shRNA constructs used in this study

Clone No.	TRC No.	Target Sequence (5'-3')
shEphB4 #73	TRCN0000001773	CAATGGGAGAGAAGCAGAATA
shEphB4 #74	TRCN0000001774	TGATCTGAAGTGGGTGACATT
shM6PR #74	TRCN0000322974	GCTCTAGTGAAGAGGCTGAAA
shM6PR #78	TRCN0000322978	CCTCATCTCACCCTTACTATT
shRab27a #1	TRCN0000380034	GAAGGAGTGGTGCGATCAAAT
shRab27a #2	TRCN0000380306	GATCTTCTCTATGATTGATAC

Table S2. List of antibodies used in Western blot

Antibodies	Source	Dilution	Provider	Catalog number
**Primary antibodies**				
ALIX	Mouse	1:1000	Cell Signaling Technology	2171
β-actin	Mouse	1:500	Santa Cruz Biotechnology	sc-8432
Calnexin	Rabbit	1:1000	Cell Signaling Technology	2679
CD63	Mouse	1:1000	Abcam	ab193349
EphB4	Mouse	1:500	Santa Cruz Biotechnology	sc-365510
GAPDH	Rabbit	1:50000	Proteintech Group	10494-1-AP
GOLM1	Mouse	1:500	Santa Cruz Biotechnology	sc-365817
GRB2	Rabbit	1:1000	Cell Signaling Technology	3972
ITGA5	Rabbit	1:1000	Cell Signaling Technology	4705
LMAN1	Mouse	1:500	Santa Cruz Biotechnology	sc-365158
M6PR	Mouse	1:500	Santa Cruz Biotechnology	sc-365196
MDK	Rabbit	1:5000	PeproTech	500-P171
Notch2	Rabbit	1:1000	Cell Signaling Technology	5732
Rab27a	Mouse	1:500	Santa Cruz Biotechnology	sc-74586
SDF4	Mouse	1:500	Santa Cruz Biotechnology	sc-393930
SRGN	Mouse	1:500	Santa Cruz Biotechnology	sc-393521
SRGN	Rabbit	1:250	Sigma-Aldrich	HPA000759
TENM-2	Sheep	1 μg/mL	R&D Systems	AF4578
TSG101	Mouse	1:500	Santa Cruz Biotechnology	sc-7964
**Secondary antibodies**				
Anti-mouse IgG, HRP-linked	Horse	1:2500	Cell Signaling Technology	7076
Anti-rabbit IgG, HRP-linked	Goat	1:2500	Cell Signaling Technology	7074
Anti-sheep IgG, HRP-linked	Donkey	1:1000	R&D Systems	HAF016
